# Determinants of Perceived Accessibility of Maternity Leave and Childcare Leave in South Korea

**DOI:** 10.3390/ijerph181910286

**Published:** 2021-09-29

**Authors:** Eun Jung Kim, Won Ju Hwang, Mi Jeong Kim

**Affiliations:** 1 School of Architecture, Hanyang University, Seoul 04763, Korea; ejkim82@hanyang.ac.kr; 2 College of Nursing Science, Kyung Hee University, Seoul 02447, Korea; hwangwj@khu.ac.kr

**Keywords:** childcare leave, determinants, maternity leave, perceived accessibility, South Korea

## Abstract

This study examined the determinants of perceived accessibility of maternity leave and childcare leave in South Korea. Although maternity leave and childcare leave are mandated in Korea, many employees are hesitant to use the policies. The purpose of this study was to empirically examine why some women are more likely than others to perceive the policies as inaccessible and to identify what those women’s characteristics are. The results revealed that nonregular workers were significantly less likely than regular workers with secure contracts to perceive the policies as accessible even though they were eligible for them. In addition, workers who worked in the private sector, did not belong to a labor union, worked in small firms, or worked long hours were significantly less likely to perceive the policies as accessible than those who worked in the public sector, belonged to a labor union, worked in large firms, or worked short hours. Further, workers with low salaries were significantly less likely than workers with high salaries to perceive the policies as accessible. The study underscores that accessibility of leave policies in Korea is significantly correlated with women’s employment status and wage level in the labor market.

## 1. Introduction

In 2020, the total fertility rate in South Korea (hereafter referred to as Korea) was 0.84, the lowest in the world [1]. Difficulties balancing work and family responsibilities have been associated with women’s reluctance to have children in Korea [2]. According to a 2009 Organisation for Economic Co-operation and Development (OECD) report, while married Korean working women spent on average 3 h per day on unpaid domestic work, men spent on average 30 min per day [3]. The Confucian patriarchal culture has placed many burdens on Korean working women, who are still considered responsible for the majority of household tasks [4]. In an effort to increase fertility among working women and promote work–family balance, the Korean government has actively expanded maternity leave and childcare leave since the 2000s [4]. However, despite the government’s effort, the fertility rate has constantly dropped in Korea, and the actual use of maternity leave and childcare leave by working women still remains relatively low [4]. According to a 2012 national report, 24% of Korean employed women who gave birth used maternity leave, and 35% used childcare leave [5].

Although maternity leave and childcare leave are mandated by the government, many Korean companies disapprove of employees taking advantage of the policies. Hence, a considerable number of Korean employees perceive maternity leave and childcare leave as inaccessible because they fear negative reprisals (i.e., being demoted to unfavorable positions, being passed over for promotions, and being laid off after their contract ends). In a 2008 report by the Korean Women’s Development Institute (KWDI), 56% and 59% of wage-earning working women reported that their companies did not provide or allow maternity leave and childcare leave, respectively [6].

Despite the low use of maternity and childcare leave in Korea, until now, to our knowledge, no study has empirically examined the reasons why some women in Korea are less likely than others to perceive maternity leave and childcare leave as accessible and to identify what those women’s characteristics are on a national scale. Kim examined 1000 working Korean mothers with preschool children from five metropolitan areas in 2011–2012 and found that intent to use family policy was influenced by work-oriented attitude, household financial status, informal (i.e., family members and neighbors) support, work sector (i.e., private sector vs public sector), and awareness and familiarity with family policies [7]. However, Kim’s findings cannot be generalizable because the study only relies on five metropolitan areas. In Korea, significant differences exist between people dwelling in metropolitan areas and rural areas (i.e., income, age, and social infrastructures). For example, income is important because it is directly related to affordability of leaves. In 2012, the annual average household income for rural residents was substantially lower than for residents dwelling in metropolitan areas (USD 30,000 vs USD 54,000 [7]. Additionally, rural areas have fewer social infrastructures such as childcare centers to help working parents with childcare and other domestic responsibilities. Hence, the goal of the present study was to address this gap in the literature. Using the 2007–2012 Korean Longitudinal Survey of Women and Families (KLoWF), the present study examined on a national scale the factors associated with working women’s perceived accessibility of maternity leave and childcare leave in Korea. Findings from this study will help policymakers to develop policies that better facilitate working women’s access to maternity leave and childcare leave in Korea.

## 2. Maternity Leave and Childcare Leave in Korea

In Korea, both maternity leave and childcare leave are mandated under the Act on Equal Employment and Support for Work-Family Balance. To be eligible for maternity leave and childcare leave in Korea, workers must have been employed at their workplaces and enrolled in the National Employment Insurance program for more than 180 days. In Korea, all companies—except for (1) unincorporated businesses with four or fewer full-time employed workers in agricultural, forestry, fishing, or hunting industries; (2) small construction companies with a yearly total construction cost of less than KRW 20 million (approximately USD 17,300); and (3) self-employed businesses—are mandated to provide the National Employment Insurance to their employees. Hence, except for the few abovementioned cases, the majority of wage-earning Korean workers should be automatically eligible for maternity leave and childcare leave if they have worked at the workplace for more than 180 days. The penalty for the company refusing to provide maternity or childcare leave is KRW 5,000,000 (USD 4500) per case, but it only applies when reported by a refused employee. Over the years, the Korean government has expanded maternity leave and childcare leave to promote work–family balance and fertility.

### 2.1. Maternity Leave

Maternity leave was originally introduced in 1953 as a paid 60-day leave policy but was extended in 2001 to 90 days in Korea. Currently, in Korea, maternity leave can be used prior to birth for up to 45 days. Working Korean women are eligible to receive full replacement wages for the first 60 days of their leave from employers and partial or full-wage replacement from the National Employment Insurance program for the remaining 30 days up to a maximum of KWR 2,000,000 (USD 1750).

### 2.2. Childcare Leave

Childcare leave was first introduced in 1987 as unpaid one-year leave for mothers with children under one year old. In 2008, this policy was revised to provide paid leave at a flat rate of KRW 500,000 (USD 450) per month and was expanded to include both mothers and fathers with children under three years old. In 2010, the leave was further expanded to include children under six years old. In 2011, the flat rate was revised from KRW 500,000 (USD 450) per month to a wage replacement equivalent to 40% of the receiver’s monthly salary within a range of KRW 500,000–1,000,000 (USD 450–900). In 2014, it was revised to include children under eight years old (or until they enter the second year of primary education). In 2018, the wage replacement rate was revised to 80% of their monthly salary within a range of KRW 700,000–1,500,000 (USD 600–1300) for the first 3 months of their leave, and 40% of their salary within a range of KRW 500,000–1,000,000 (USD 450–900) for the remaining 9 months. Additionally, from 2018, to promote mothers’ return to work, only a partial 75% of the benefit is provided during the leave period and the remaining 25% of the benefit is paid as a lump sum 6 months after mothers return to work.

Employees who are unable to take full-time one-year absence may use reduced working hours as an alternative to childcare leave. To be eligible for such leave, employees must work for a minimum of 15 h per week and a maximum of 35 h per week and may use the leave for up to a maximum of 2 years in one or two blocks before the child’s eighth birthday.

### 2.3. Low Uptake of Maternity Leave and Childcare Leave

In comparison with other OECD countries, the overall length of statutory maternity and childcare leave support in Korea is quite extensive. Mothers in Korea can take up to 65 weeks of paid leave in total, which is longer than the OECD average of 55 weeks [8]. However, as mentioned above, the uptake rate of maternity leave and childcare leave remains low in Korea. For children born in 2017, only about 23% of mothers used maternity leave benefits, and 11% of them worked in the civil public sector or teaching professions [9]. Regarding childcare leave, for children born in 2018, only about 30% of parents claimed the benefit, and 53% of them were government officials or teachers (in schools or universities). Compared with other OECD countries, this uptake is relatively low. For example, in Germany in 2016, approximately 94 mothers and 35 fathers claimed childcare leave for every 100 births [9].

## 3. Literature Review of Determinants of Maternity Leave and Childcare Leave Accessibility

A possible reason for the low usage of maternity leave and childcare leave is that employees believe that access to such provisions is restricted [10]. The literature explains this as a gap between “policy and practice” [11,12,13]. Employees might not report that leave policies are accessible to them even if they are available at their workplaces for several reasons. First, companies may offer certain benefits only to specific groups of employees (i.e., uneven coverage within a workplace). Second, employees may not be aware of the benefits to which they are entitled. Lastly, employees may feel that these policies are inaccessible because of various barriers, such as financial constraints, impracticalities, and fear of reprisals [10].

However, in Korea, since maternity leave and childcare leave are statutory, companies do not have the discretion to choose to whom to provide provisions. In addition, because maternity leave and childcare leave have been implemented since 1953 and 1987, respectively, it is unlikely that employees’ reason for not using the policies is the lack of awareness of the programs in Korea. Dulk and Peper argued that employees must have a sense of entitlement for them to access and use the policies and contended that social atmosphere and institutional features pose as barriers or enablers to employees’ sense of entitlement to policies [14]. Since there is a lack of studies on policy accessibility and its determinants in Korea, the present study used studies from Western countries (e.g., the Netherlands, United Kingdom, and United States) [10,14,15] to understand the mechanisms behind why some women are less likely than others to perceive the leave policies as accessible. We believe it would be interesting to examine whether in Korea the results would be different. Compared with Western countries, Korea has a strong collectivist workplace culture, which places “team/group” above “individual” [16]. On the other hand, in Western cultures, there is more focus on the individual and that collective goods are to serve individual rights, not vice versa. Hence, personal and family time is a priority; however, in Korea, priority is often given to work [16]. Such differences may yield different results.

### 3.1. Public Sector Employees

Dulk and Peper’s arguments are corroborated by findings from Budd and Mumford’s examination of the relationship between institutional context (i.e., norms, regulations, and social expectations) and employees’ perceived access to policies in the United Kingdom [10,14]. They posited that some employees are more sensitive to institutional pressure than other employees, based on their visibility and influence in society, and found that public sector employees were more likely to feel entitled to maternity and childcare leave and to perceive the policies as accessible because they were more visible to the public and sensitive to government policies. Hence, in this study, we hypothesized that:

**Hypothesis** **1** **(H1)**: *Women working in the public sector are more likely to perceive maternity leave and childcare leave as accessible in Korea.*

### 3.2. Labor Union Members

Budd and Mumford also discovered that more than those who did not belong to labor unions, employees who belonged to labor unions were more likely to perceive leave policies as accessible because they had stronger collective bargaining power to negotiate benefits and, having been educated through union letters and workshops, had greater awareness of maternity leave and childcare leave, and thus felt a stronger sense of entitlement to these policies [10]. In Korea, although a majority of the people are aware of the existence of the policies, some employees may not be aware that they are also eligible for the policies. Hence, those who belong to labor unions are more likely to be aware of their entitlements and have greater bargaining power to fight against employers who refuse to provide maternity leave or childcare leave. Hence, in this study, we hypothesized that:

**Hypothesis** **2** **(H2)**: *Working women belonging to labor unions are more likely to perceive maternity leave and childcare leave as accessible in Korea.*

### 3.3. Nonregular Temporary Workers

Budd and Mumford also discovered that regular employees were more likely than nonregular workers to perceive leave policies as accessible because they had greater job security [10]. In Korea, nonregular workers are often deprived of their right to apply for maternity and childcare leave because of the workplace culture and regulations that forbid them from taking the leave or simply do not acknowledge their rights. Although in Korea, the law stipulates that employees are automatically eligible for maternity leave and childcare leave, regardless of their contract status, as long as they are employed for more than 6 months at their workplaces. However, nonregular workers may not know this or feel they cannot use the policies. According to a survey conducted by the KWDI in 2009, about half of nonregular workers did not apply for maternity leave, and about a third of those who did not apply reported that they did not apply because their workplace regulations forbade them from taking leave, which is not only discriminatory but also illegal [17]. However, nonregular workers dared not challenge the companies’ regulations for fear of losing their work contract in the subsequent year. Hence, we hypothesized that:

**Hypothesis** **3** **(H3)**: *Women who are regular workers are more likely to perceive maternity leave and childcare leave as accessible in Korea*.

### 3.4. Company Size

It has also been reported that smaller firms are more likely than big companies in Korea to have work regulations that forbid workers from using leave [18]. This is possibly because the first two months of maternity leave are paid for by the employer, and smaller firms may have less financial capability to support such costs. In addition, it was reported that a significant share of women in Korea prefer not to take the leave in order to avoid penalizing their coworkers, because companies often do not fill temporary vacancies [19]. Smaller firms are less likely than bigger companies to have the human and financial resources to fill the vacancies when employees take leave; therefore, they are more likely to have workplace cultures or internal regulations that hinder employees from using maternity and childcare leave. Hence, we hypothesized that:

**Hypothesis** **4** **(H4)**: *Women who work in small companies are less likely to perceive maternity leave and childcare leave as accessible in Korea.*

### 3.5. Gendered-Discriminatory Workplace and Long Working Hours

The internal culture of the organization is argued to also influence the extent to which employees feel entitled to leave policies [14]. There is mounting evidence that workplace culture substantially affects workers’ perceived access to and use of maternity and childcare leave [20]. Fried discovered that in workplaces where the workforce composition was a “gendered pyramid, with the greatest number of women at the bottom and increasingly fewer women as one moves up to the top”, long working hours were valued, the workplaces were often permeated by a male-defined culture (i.e., competition, aggression, and singular focus on career, usually at the expense of family), and employees tended to perceive leave polices as less accessible [15] (p. 40). In Korea, work culture is marked by long working hours (46.8 h per week, compared with the OECD average of 42 h; OECD 2019) and strong work ethics, primarily dominated by male managers [4]. According to a survey conducted by Job Korea in 2010, more than half of the 1623 female workers interviewed reported that it was difficult to use maternity and childcare leave because of the pressure from their workplaces [21], and according to a survey conducted by Samsung Economic Research Institute, 42% of female respondents reported that they feared receiving low evaluations during or after they returned from maternity and childcare leave [22]. Hence, in this study, we hypothesized that:

**Hypothesis** **5a** **(H5a)**: *Women who work in a gender-discriminatory workplace culture are less likely to perceive maternity leave and childcare leave as accessible in Korea*.

**Hypothesis** **5b** **(H5b)**: *Women who work long hours are less likely to perceive maternity leave and childcare leave as accessible in Korea*.

### 3.6. Managerial Positions

Lastly, Fried also discovered that neither men nor women at the upper levels used leave policy, and middle-level managers used it sparingly in the United States [15]. The largest cohort of leave-takers comprised female non-managers. In addition, according to the 2010 Korean Women Manager Panel, only 36% of managers applied for childcare leave, and among those who did not apply, one-fourth responded that they could not apply for the leave because, according to their workplace culture, it is customary not to apply [23]. In a similar line of reasoning, the present study expected women workers with high salaries to be less likely to perceive maternity leave and childcare leave as accessible than workers with lower salaries because they have greater work responsibilities and higher peer pressure. In this study, because of data limitations, salary was used as a proxy variable to measure career-level position. The literature indicates that there is a significant positive correlation between salary and career [24]. Moreover, considering that only a partial rate of the salary is paid during the leave period, for employees with higher salaries, the opportunity cost of leaving work to take care of children will be higher, and hence, they may be less inclined to take maternity and childcare leave than employees’ with lower salaries. Hence, we hypothesized that:

**Hypothesis** **6** **(H6)**: *Women with higher wages are less likely to perceive maternity leave and childcare leave as accessible in Korea.*

## 4. Methodology

### 4.1. Data

The KLoWF was used for this study. The KLoWF uses a multi-stage, stratified clustered design and surveys 9997 women aged 19 to 64 who live in 9068 households across the nation. The survey was first conducted in 2007 and subsequently in 2008, 2010, and 2012. The KLoWF is an unbalanced panel survey and refreshment samples were added to supplement for attrition. To treat for possible attrition biases, the present study used post-stratification survey weights provided by the KLoWF. Post-stratification weights adjust attrition by assuming that dropouts occur randomly within weighting classes defined by observed variables that are associated with dropouts [25].

### 4.2. Analytical Sample

In this study, to ensure that our analytical sample was eligible for maternity and childcare leave, our analytical sample included married, wage-earning, full-time working women of childbearing age (16–49) and those who had worked at their workplaces for more than 6 months, excluding those who were self-employed and worked in agricultural, forestry, fishing, or hunting industries with fewer than four full-time employees. We were unable to exclude those working in small construction companies with a yearly total construction cost of less than KRW 20 million because of data limitations: however, we posit that this number would have been minimal because the percentage of women working in construction is very small in Korea.

Based on these criteria, a total sample of 4559 observations (wave 2007 = 1174, wave 2008 = 1154, wave 2010 = 1118, wave 2012 = 1113) was examined. To treat for possible biases due to repeated measures, random effects modeling was used in this study, which is further explained in Section 4.4.

### 4.3. Measures

#### 4.3.1. Outcome Variables

The outcome variables for this study were working women’s perceived access (whether employees could actually use the policies if they wished) to maternity leave and childcare leave. Accessibility was measured as a binary response (yes or no).

#### 4.3.2. Predictor Variables

Employment status (regular or nonregular worker), work sector (public or private), and belonging to a labor union (yes or no) were measured as dichotomous variables. Company size was measured as a categorical variable (1 = below 5 employees, 2 = 5–9 employees, 3 = 10–29 employees, 4 = 30–99 employees, 5 = more than 100). Average work hours per day and logged monthly salary were measured as a continuous variable. Gender-discriminatory workplace culture was measured using the following six items: (1) “If candidates have similar qualifications for appointment, men are preferred to women”; (2) “Even with identical or similar careers, male workers are promoted faster than female counterparts”; (3) “Even in identical or similar positions, male workers receive higher wages and bonuses than female workers”; (4) “Duties are fixed or customarily divided between men and women”; (5) “Even with similar duties, men have more opportunities to receive education and training than women”; and (6) “In case of restructuring, female workers are more likely to be forced to quit”. The KLoWF reports the six items using a 4-point Likert scale (1: nondiscriminatory to 4: discriminatory), and in this study, the items were summed and averaged to measure gender-discriminatory workplace culture.

#### 4.3.3. Control Variables

A range of demographic variables was controlled in this study. Participants’ age, average hours spent on unpaid household chores, and logged yearly household income were measured as continuous variables and controlled. Number of children was measured as a categorical variable (0 = none, 1 = one child, 2 = two children, 3 = three or more children). Education attainment was measured as a 4-point ordinal scale (1 = middle school degree or below, 2 = high school degree, 3 = two-year community college degree, 4 = four-year bachelor’s degree or master’s or higher). We have summarized the variables in Table 1. 

### 4.4. Modeling

Since the KLoWF is a longitudinal panel survey, a pooled ordinary least squares (OLS) regression model with year dummies may yield biased results because of potential omitted variables (i.e., unobserved heterogeneity), such as social and cultural attitudes. Considering that leave policies are closely related to contextual influences such as social and cultural attitudes toward working women, it is important to control for such variables [26].

Both a fixed effects model and random effects model can account for unobserved heterogeneity [27]. The purpose of both fixed and random effects estimators is to consider treatment effects whilst controlling for unobserved individual-specific effects. In the model below, this is represented by α_i_

Y_it_= βX_it_ + α_i_ + e_it,_

where,

- Y_it_ is the dependent variable (i = individual, t = time);
- X_it_ is the independent variable;
- β is the coefficient;
- α_i_ is the individual-specific intercept;
- e_it_ is the error term.

In a fixed effects modeling, unobserved heterogeneity is accounted for by allowing each individual to have its own intercept (α_i_). In fixed effects modeling, each individual is assigned a specific intercept value (hence, the name fixed effects), and by doing so, we eliminate all individual-specific effects, including both observable and unobservable individual-level factors and focus solely on the impact of variables that vary over time. A disadvantage, however, is that time-invariant variables cannot be estimated. Since individual-level intercepts are essentially separate independent variables in a fixed effects model, we assume that the intercepts are correlated with the independent variables.

In a random effects model, individual-specific effects, including unobservable factors, are captured by the composite error terms (α_i_ + e_it_), which assumes that individual intercepts (α_i_) are randomly distributed (hence, the name random effects), unlike in the fixed effects model, which assigns fixed specific values. In order to capture individual heterogeneity, random effects modeling estimates error variances specific to cross-sectional units [27]. Since in the random effects model, the variation across individuals is random, we assume that the intercepts are uncorrelated with the independent variables. Hence, in a random effects model, a one-unit increase in independent variable “X” may have two meanings: (1) differences between individuals when there is a unit difference in “X” between them (i.e., between effect) and (2) differences within an individual when “X” increases by one (i.e., within effect). The random effects model estimates that the two effects are the same [25].

Since this study was interested in estimating the overall effect of determinants of perceived accessibility of maternity and childcare leave between and within individuals, the random effects model was selected. Hausman specification tests were conducted to test whether fixed effects or random effects modeling was a better model fit, and the results confirmed the appropriateness of using random effects modeling over fixed effects modeling. Here, we used a random effects logistic model since our dependent variables were binary variables.

Further, we controlled for possible sample selection bias (i.e., bias due to a flaw in the sample selection process, where a subset of the data is systematically excluded due to a particular attribute [26]) by using STATA 16 extended regression model (ERM) analysis (see Appendix A Table A1). Since in our study perceived accessibility of maternity leave and childcare leave was only observed among working women, there was the possibility that working women may have had attributes that were significantly different and non-random from women not participating in the labor market. That is, women who were less likely to perceive maternity leave and childcare leave as accessible may have been less likely to join the labor market from the start (i.e., selection bias). However, the ERM results showed that no significant sample selection bias existed in our model; hence, in this study, we used the more parsimonious random effects logistic model as our final model.

In addition, if we were to run a pooled ordinary least squares regression model with year dummies, the results may have been biased because of autocorrelation due to repeated measures (i.e., correlation between residuals at a given wave and the ones for the previous one) [26]. To control for the possible existence of autocorrelation, we used cluster-robust standard errors in all the models [26].

## 5. Results

Table 2 presents the accessibility rates by time. The results show that approximately 26% and 21% of working women reported that they felt they could use maternity leave and childcare leave, respectively, if they wished to. Over the years, despite the government’s efforts, a lower percentage of people have perceived the leave policies as accessible.

Table 3 presents the unadjusted bivariate results between policy accessibility and the predictors and covariates. First, with regard to maternity leave, while 60% of regular workers perceived maternity leave as accessible, less than 5% of nonregular workers perceived maternity leave as accessible (*p* < 0.001). Likewise, while the majority (72%) of workers who belonged to labor unions perceived maternity leave as accessible, only 18% of workers who did not belong to labor unions perceived maternity leave as accessible (*p* < 0.001). In addition, women who worked in the public sector (61%) were approximately 3 times more likely to perceive maternity leave as accessible than those who worked in the private sector (23%; *p* < 0.001). Moreover, workers who worked in larger companies were also significantly more likely to perceive maternity leave as accessible (*p* < 0.001), and those who worked longer hours were more likely to perceive maternity leave as accessible (*p* < 0.001). Workers who worked in more gender-discriminatory workplaces were more likely to perceive maternity leave as accessible, but this was not statistically significant. Lastly, working women whose salary was higher were significantly likely to perceive maternity leave as accessible (*p* < 0.001).

Second, with regard to childcare leave, similarly, regular workers (42%) were significantly (*p* < 0.001) more likely to perceive the policy as accessible than nonregular workers (3%). Likewise, those who belonged to labor unions (63%) were significantly (*p* < 0.001) more likely to perceive childcare leave as accessible than those who did not belong to labor unions (13%). Working women who worked in the public sector (84%) were approximately 2 times more likely to perceive childcare leave as accessible than those in the private sector (43%). In addition, those working in larger companies were significantly more likely to perceive childcare leave as accessible (*p* < 0.001). The results show that those who work in gender-discriminatory workplaces were significantly more likely to perceive childcare leave as accessible (*p* < 0.001), while those who worked longer hours were also more likely to perceive childcare leave as accessible, but this was not statistically significant. Lastly, working women whose salary was higher were significantly likely to perceive childcare leave as accessible (*p* < 0.001).

Table 4 presents the random effects logistic model results after controlling for other model covariates. First, regarding maternity leave, the results indicate that the odds of public sector workers perceiving maternity leave as accessible were 2.87 times higher than for private sector workers (*p* < 0.001). Likewise, those who were labor union members were 2.58 times more likely to perceive maternity leave as accessible than non-labor union members (*p* < 0.001). The odds of regular workers perceiving maternity leave as accessible were 12.25 times higher than nonregular workers (*p* < 0.001). In addition, those working in larger companies were significantly more likely to perceive maternity leave as accessible (*p* < 0.001), and long working hours were reported to significantly decrease the odds of perceiving maternity leave as accessible (*p* < 0.01). The odds of perceiving maternity leave as accessible increased by 8.19 times with every additional 1% increase in monthly salary (*p* < 0.001). However, gender-discriminatory workplaces did not have significant associations with working women’s perception of maternity leave accessibility.

Second, regarding childcare leave, results similarly indicate that the odds of public sector workers perceiving childcare leave as accessible were 3.72 times higher than for private sector workers (*p* < 0.001). Likewise, those who were labor union members were 2.57 times more likely to perceive childcare leave as accessible than non-labor union members (*p* < 0.001). The odds of regular workers perceiving childcare leave as accessible were 13.39 times higher than for nonregular workers (*p* < 0.001). Further, those working in larger companies were significantly more likely to perceive childcare leave as accessible (*p* < 0.001), and long working hours were reported to significantly decrease the odds of perceiving childcare leave as accessible (*p* < 0.001). The odds of perceiving childcare leave as accessible increased 7.32 times with every 1% increase in monthly salary (*p* < 0.001). Gender-discriminatory workplaces did not have significant associations with the perception of childcare leave accessibility.

We summarize the results in comparison with our initial hypotheses in Table 5. Contrary to our hypotheses, gender-discriminatory workplace culture was not significantly associated with working women’s perception of access to both maternity leave and childcare leave. Moreover, while we initially hypothesized that those with higher salaries would be less likely to perceive the leave policies as accessible, the results show that, on the contrary, they were more likely to perceive maternity leave and childcare leave as accessible in Korea, which we believe is because in Korea, salary is not only associated with managerial positions but also with company characteristics. Korea’s labor market is highly polarized by company size [28]. Even among similar industries, large companies usually provide much higher salaries with better fringe benefits than smaller companies, which has been accelerated since the Asian Economic Crisis of 1997 [29].

## 6. Discussion

This study examined the determinants of perceived access to maternity leave and childcare leave among Korean wage-earning full-time working women of childbearing age. First, the results show that despite the government’s efforts to expand leave policies, the majority of working women perceived the leave policies as inaccessible. On average, only 26% and 21% of the sample reported that they could use maternity leave and childcare leave, respectively, if they wished to. Second, the results indicate that those who worked in the public sector, belonged to labor unions, worked in big companies, were regular workers, and earned high salaries were more likely to perceive maternity leave and childcare leave as accessible, whereas those who worked long hours were less likely to perceive maternity leave and childcare as accessible.

### 6.1. Limitations

Before discussing the study implications, the limitations require consideration. First, the study focused on examining the determinants of maternity leave and childcare leave quantitatively. Further qualitative studies are warranted to understand the complex mechanisms behind working women’s perception of maternity leave and childcare leave. Second, the present study examined the determinants of women’s perceived accessibility and not the actual usage. Different factors may play a role in women’s use of maternity leave and childcare leave. For example, women in higher positions may have greater authority and thus may perceive leave policies as being more accessible; however, they may voluntarily choose to not use these policies because they are more work-oriented. Further studies are needed that compare the determinants of perceived accessibility and actual usage of leave policies. Third, since in this study we did not lag (i.e., create variables that were one period behind in time) our predictor variables, we cannot guarantee a causal relationship. We decided not to lag our predictor variables because we were interested in examining how the respondents’ current status was associated with their current perception of maternity leave and childcare leave, as a result, strictly speaking, we examined association instead of causality. Nonetheless, based on Dulk and Peper [14] and other previous studies [10,11,12,13,15], we assume that the respondents’ current status affected their perceived accessibility of maternity leave and childcare leave. Fourth, due to data limitations, salary was used as a proxy variable to measure career-level position. We acknowledge that salaries differ in particular sectors of the economy, size classes of cities, regions, and occupations. However, we believed it would still be interesting and meaningful to examine salary. Future studies are called for with improved data.

Despite these limitations, the study has notable strengths. The study is the first Korean study to empirically examine the determinants of perceived accessibility of maternity leave and childcare leave using a national sample of working women. Restricted accessibility has been a common recurring issue not only in Korea but also internationally; however, there are very few empirical studies on why some employees are less likely to perceive maternity leave and childcare leave as accessible. This paper contributes to understanding the determinants of working women’s perceived access to maternity leave and childcare leave, and informs policymakers of factors that could help improve policy accessibility, and possibly usage.

### 6.2. Policy Implications

Our results have important policy implications. First, in our study, only one in four working women and one in five working women perceived maternity leave and childcare leave as accessible, respectively. A possible reason may be because currently, penalties against employers who violate the requirement to provide leave policies are weak in Korea [30]. For instance, companies that refuse to provide leave to employees are penalized only KRW 5,000,000 (USD 4500) when reported by the refused employee [30]. Hence, the authors believe stricter penalties and heightened enforcement should be implemented for employers who do not follow the law in order to increase accessibility. In addition, it is important to create a workplace culture where employees feel they can use the policies without the fear of reprisal.

Second, among all the predictors, regular work had the most significant association with women’s perceptions of the accessibility of maternity leave and childcare leave. Regular workers perceiving maternity leave and childcare leave as accessible was 12 times and 13 times higher than for nonregular workers, respectively. Considering that all the samples analyzed in this study had worked for more than 180 days and were eligible to apply for the policies, this disparity is striking. Although nonregular workers are often involved in the same or similar tasks in the workplace as regular workers in Korea, their terms (i.e., wages) and employment conditions (i.e., exclusion from company benefits) tend to fall below those of regular workers [28]. Nonregular employment contracts became a popular form of contract in Korea during the 1997 Asian Economic Crisis to avoid providing full-time job security and welfare entitlements, which was justified as a necessity to overcome the crisis and to remain economically solvent at the time. However, it remained popular even after the crisis ended [28]. It is likely that nonregular workers, in fear of losing their jobs or contracts in subsequent years, will not request leave policies as frequently as regular workers, whose job security is protected by the Labor Standard Act and Social Security Act. Under these Acts, regular workers cannot be subject to a definite employment termination date unless they reach the mandatory retirement age or have committed substantial misconduct. Although it is illegal to deprive nonregular workers of maternity leave and childcare leave, some companies forbid (both directly and indirectly) nonregular workers from taking leave in their regulations or contracts [31]. In 2016, 40% of total Korean female workers were nonregular workers [32]. As a result, a possible reason for Korea’s low policy usage rate may be the high number of female nonregular workers and their inability to access maternity leave and childcare leave, in addition to the government’s failure to properly regulate discrimination against nonregular workers in the labor market.

Third, the results show that those who worked in the public sector were 2.9 times and 3.7 times more likely to perceive maternity leave and childcare leave as being accessible, respectively. This is probably because the public sector is more sensitive to government policies, and hence it is a workplace environment that is more accepting of and accommodating to employees using leave policies. Statistics show that in 2017, among mothers who used maternity leave, approximately one-third of them worked in the public sector [9], manifesting that, as hypothesized, in Korea those who work in the public sector are more likely to perceive leave policies as accessible.

Fourth, the results indicate that, in contrast to Western studies and our initial hypothesis, women with higher salaries were significantly more likely to perceive maternity leave and childcare leave as being accessible in Korea. We believe this is because, in Korea, as previously mentioned, higher-earnings are associated not only with managerial positions but also with company characteristics. Korea’s labor market is highly polarized between those employed in small companies with low wages and those employed in large companies with high salaries and good fringe benefits [28]. Hence, those earning high salaries are more likely to be employed in large companies. However, the problem lies in the fact that a substantial number of Korean working women are low-wage workers, earning less than two-thirds of the median-salary wage earners [33]. In 2014, 38% of Korean working women were low-wage workers earning less than KRW 6712 (USD 5.84) an hour [31]. Although childcare leave is paid in Korea, it currently only covers a maximum of 40% of a mother’s monthly salary, within the range of USD 450–900. Working mothers, especially mothers with low earning capacity, hence are likely not able to take leave or afford a lower income. Thus, in this study, we suggest expanding the benefit amounts to be more generous to increase accessibility and use of maternity leave and childcare leave in Korea.

Lastly, long working hours were negatively associated with working women’s perceived accessibility of maternity leave and childcare leave. In 2019, a Korean worker worked on average 1967 h per year, which is 241 h more than the OECD average and the highest among the OECD countries [34]. Korean work culture emphasizes strong a work ethic and long working hours, which makes it difficult to reconcile work and family lives [35]. What we found in this study was that it is important to decrease working hours in order to increase accessibility and usage of maternity leave and childcare leave.

## 7. Conclusions

In summary, the present study is the first to empirically examine the determinants of Korean working women’s perceptions of the accessibility of maternity leave and childcare leave in Korea using a national sample. The study discovered that employment status (regular vs nonregular worker), working in the public sector, belonging to a labor union, company size, and salary were the important determinants of perceived accessibility of leave policies in Korea. However, a significant number of Korean working women are nonregular low-wage workers who work in small companies without labor unions. Hence, we suspect this may be one of the reasons for Korea’s low leave usage and probably Korea’s very low fertility rate. Whilst the period of the so-called “demographic transition” involves a significant decline in fertility linked with economic development, this negative relationship has weakened, and some countries such as France and Sweden have experienced an increase in fertility rates since the late 1990s, while their economies continue to grow [9]. On the other hand, East Asian countries such as Singapore, Korea, and Taiwan experienced a continued drop in their fertility, hovering around 1.0–1.2 children per woman—the lowest low fertility (i.e., total fertility rate at or below 1.3) [36]. Reports show that countries that experienced an increase in their fertility had relatively high work–family balance and gender-equality [9,36]. For example, Sweden offered a comprehensive support system for working parents through a combination of generous leave policies and widely available childcare services. The uptake rate of maternity leave and childcare leave is also high in Sweden [37]. Hence, policymakers interested in increasing fertility rates should therefore consider addressing these issues and try to improve the accessibility and use of maternity leave and childcare leave. Laws that prohibit discrimination against nonregular workers and policies that promote women’s status in the labor market should be considered.

## Figures and Tables

**Table 1:** Summary of the variables.

Variables		Measures
Outcome	Perceive maternity leave as accessible	No (=0)/Yes (=1)
	Perceive childcare leave as accessible	No (=0)/Yes (=1)
Predictor	Employed in public sector	Private (=0)/Public (=1)
	Labor union member	No (=0)/Yes (=1)
	Regular worker	Nonregular (=0)/Regular (=1)
	Company size	Below 5 (=1)/5–9 (=2)/10–29 (=3)/30–99 (=4)/ more than 100 (=5)
	Gender-discriminatory workplace	Six-item Likert scale (1–4)
	Working hours	Average daily working hour (continuous)
	Monthly wage	Logged after-tax wage KRW 10,000 (continuous)
Control	Age	Age (continuous)
	Household chores	Average hours spent on household chores (continuous)
	Number of children	0 (=0)/1 (=1)/2 (=2)/3 or more (=3)
	Household income	Logged yearly after-tax household income (continuous)
	Education	(1 = middle school degree or below, 2 = high school degree, 3 = two-year community college degree, 4 = four-year bachelor’s degree or master’s or higher)

**Table 2:** Maternity leave and childcare leave accessibility time.

	Wave 2007	Wave 2008	Wave 2010	Wave 2012	Overall
Maternity leave	32.2%	26.3%	23.1%	21.5%	25.8%
Childcare leave	28.4.%	23.3%	19.7%	13.9%	21.4%

**Table 3:** Bivariate analyses (*N* = 4559).

Frequency (%)	Maternity Leave			Childcare Leave		
	Not Accessible	Accessible	*x*^2^ (*df*)	Not Accessible	Accessible	*x*^2^ (*df*)
**Employment type**			1129.7 (1) ***			904.4 (1) ***
Regular worker	49.10%	50.90%		58.90%	41.10%	
Nonregular worker	95.20%	4.80%		97.20%	2.80%	
**Labor union**			902.8 (1) ***			921.8 (1) ***
No	81.80%	18.20%		87.10%	12.90%	
Yes	28.30%	71.70%		37.30%	62.80%	
**Work sector**			421.9 (1) ***			548.5 (1) ***
Private	77.00%	23.10%		84.00%	16.00%	
Public	38.80%	61.20%		43.00%	57.00%	
**Company size**			837.2 (4) ***			752.1 (4) ***
5 below	94.90%	5.10%		97.40%	2.60%	
5–9	85.90%	14.10%		91.50%	8.50%	
10–29	74.50%	25.50%		80.90%	19.10%	
30–99	52.40%	47.60%		58.30%	41.70%	
100 or more	42.20%	57.80%		53.80%	46.20%	
**Education attainment**			765.1 (3) ***			736.4 (3) ***
Middle school or below	98.50%	1.50%		98.60%	1.40%	
High school	87.60%	12.40%		91.40%	8.60%	
2-year community collage	64.8.%	35.20%		79.10%	20.90%	
4-year college or above	47.30%	52.70%		53.60%	46.40%	
**Number of children**			164.6 (3) ***			111.9 (3) ***
0	62.30%	38.70%		71.80%	28.20%	
1	56.10%	43.90%		65.60%	34.40%	
2	75.60%	24.40%		80.60%	19.40%	
3 or more	82.90%	17.10%		87.00%	13.00%	
***Mean* ^1^**	**Not Accessible**	**Accessible**	***t*-Test (*df*)**	**Not Accessible**	**Accessible**	***t*-Test (*df*)**
Gender-discriminatory workplace culture ^2^	2.9 (0.1)	3.1 (2.9 × 10^−2^)	−2.0 (7914) *	2.8 (0.1)	3.1 (2.8 × 10^−2^)	−3.7 (7914) ***
Average daily working hours	6.8 (0.5)	7.2 (0.5)	−3.5 (7868) ***	6.8 (0.1)	7.1 (0.1)	−1.7 (7681)
Monthly salary (KRW 10,000)	111.3 (1.5)	239.6 (3.5)	−58.3 (7814) ***	116.5 (1.6)	255.3 (3.8)	−56.6 (7629) ***
Age	40.6 (0.2)	36.4 (0.2)	27.8 (7914) ***	40.2 (0.2)	37.6 (0.3)	23.1 (7727) ***
Average daily unpaid housework minutes	196.1 (10.2)	242.1 (10.8)	−11.2 (6166) ***	201.0 (3.7)	234.3 (8.6)	−8.6 (6069) ***
Yearly household income (KRW 10,000)	4197.1 (240.5)	6207.5 (115.2)	−29.5 (7781) ***	4291.2 (57.4)	6419.9 (120.6)	−27.9 (7596) ***

Note: Values are weighted; ^1^ standard errors in parentheses; ^2^ Likert scale 1: nondiscriminatory to 4: discriminatory; * p < 0.05, *** *p* < 0.001.

**Table 4:** Multivariate logistic random effects model for determinants of leave accessibility.

	Maternity Leave	Childcare Leave
	OR (95% CI)	OR (95% CI)
Public sector	2.87 (1.91, 4.32) ***	3.72 (2.39, 5.78) ***
Labor union	2.58 (1.79, 3.72) ***	2.57 (1.80, 3.68) ***
Regular worker	12.25 (7.85, 19.11) ***	13.39 (8.11, 22.11) ***
Company size (ref: below 5)		
5–9 employees	2.28 (1.33, 3.91) **	2.20 (1.16, 4.17) *
10–29 employees	4.58 (2.65, 7.91) ***	4.95 (2.69, 9.11) ***
30–99 employees	6.30 (3.63, 10.93) ***	6.63 (3.60, 12.21) ***
100 or more employees	19.11 (10.50, 34.77) ***	14.30 (7.50, 27.23) ***
Gender-discriminatory workplace culture	1.25 (0.60, 2.09)	1.20 (0.53, 2.30)
Work hour	0.92 (0.84, 0.99) **	0.83 (0.76, 0.92) ***
Logged salary	8.19 (5.39, 1.01) ***	7.32 (4.69, 11.44) ***
Age	0.87 (0.84, 0.90) ***	0.90 (0.87, 0.93) ***
Number of children (ref: 0)		
1	1.68 (0.89, 3.17)	1.71 (0.88, 3.32)
2	1.29 (0.70. 2.37)	1.25 (0.66, 2.37)
3 or more	1.61 (0.78, 3.32)	1.73 (0.79, 3.79)
Unpaid housework hour	1.00 (0.99, 1.00)	1.00 (0.99, 1.00)
Logged household income	1.20 (0.99, 1.72)	1.09 (0.74, 1.58)
Education (ref: middle school or below)		
High school degree	6.54 (1.73, 24.64) **	2.52 (0.87, 7.27)
2-year community college	10.29 (2.65, 39.90) **	2.75 (0.89, 8.49)
4-year college or higher	11.58 (2.99, 44.75) ***	5.50 (1.82, 16.54) **
Constant	1.03 × 10^−6^ (3.98 × 10^−9^, 2.64 × 10^−5^) ***	3.43e^−6^ (1.32 × 10^−7^, 8.89 × 10^−5^)
*Sigma_u*	1.25 (Robust SE: 0.16)	1.37 (Robust SE: 0.16)
*rho*	0.32 (Robust SE: 0.05)	0.36 (Robust SE: 0.05)

* *p* < 0.05, ** *p* < 0.01, *** *p* < 0.001.

**Table 5:** Summary of the results.

Predictors	Hypotheses	Results
Public sector	Positively significant	Positively significant
Labor union	Positively significant	Positively significant
Regular worker	Positively significant	Positively significant
Large firm size	Positively significant	Positively significant
Gender-discriminatory workplace culture	Negatively significant	Insignificant
Long working hour	Negatively significant	Negatively significant
High Salary	Negatively significant	Positively significant

## Data Availability

Publicly available datasets were analyzed in this study. The data can be found here: https://klowf.kwdi.re.kr/portal/eng/dataSet/rdssListPage.do?phDivCd=P (accessed on 5 March 2021).

## Appendix A

**Table A1:** Extended Regression Model (ERM) probit analysis controlling for selection bias.

	Maternity Leave	Childcare Leave
	Coeff. (Robust SE)	Coeff. (Robust SE)
Public sector	0.48 (0.12) ***	0.59 (0.10) ***
Labor union	0.49 (0.12) ***	0.50 (0.09) ***
Regular worker	1.05 (0.19) ***	1.02 (0.14) ***
Company size (ref: below 5)		
5–9 employees	0.36 (0.12) **	0.32 (0.11) **
10–29 employees	0.72 (0.16) ***	0.70 (0.13) ***
30–99 employees	0.81 (0.17) ***	0.78 (0.14) ***
100 or more employees	1.26 (0.25) ***	1.09 (0.17) ***
Gender-discriminatory workplace culture	0.25 (0.60)	0.20 (0.53)
Work hour	−0.04 (0.01) *	−0.07 (0.02) ***
Logged salary	0.88 (0.19) ***	0.82 (0.13) ***
Age	−0.04 (0.01) *	−0.03 (0.01) **
Number of children (ref: 0)		
1	0.22 (0.14)	0.23 (0.13)
2	0.09 (0.11)	0.11 (0.12)
3 or more	0.17 (0.15)	0.21 (0.14)
Unpaid housework hour	2.78 × 10^−4^ (1.73 × 10^−4^)	−8.19 × 10^−5^ (1.63 × 10^−4^)
Logged household income	0.88 (0.08)	0.05 (0.07)
Education (ref: middle school or below)		
High school degree	0.85 (0.28) *	0.56 (0.22) *
2-year community college	1.03 (0.30) **	0.57 (0.23) *
4-year college or higher	1.04 (0.30) **	0.80 (0.23) **
constant	−7.10 (0.69) ***	−6.52 (0.63) ***
**Selected equation**		
Education (ref: middle school or below)	−0.02 (0.04)	−0.01 (0.04)
High school degree	0.01 (0.05)	−0.01 (0.05)
2-year community college	0.01 (0.04)	0.01 (0.04)
4-year college or higher		
Age	0.24 (0.01) ***	0.25 (0.01) ***
Age^2^	−3.09 × 10^−3^ (0.01) ***	−3.12 × 10^−3^ (0.01) ***
Log household income	0.15 (0.01) ***	0.15 (0.01) ***
Have a preschool child	−0.20 (0.02) ***	−0.20 (0.02) ***
Constant	−6.86 (0.30) ***	−6.91 (0.29) ***
Corr (perceived accessibility, work)	0.44 (0.48)	0.46 (0.28)

* *p* < 0.05, ** *p* < 0.01, *** *p* < 0.001.
